# The role of leptospiral proteins in immune evasion and inflammatory response stimulation in HEK293T cell monolayers

**DOI:** 10.3389/fimmu.2025.1689798

**Published:** 2025-12-09

**Authors:** Igor R. M. Silva, Aline F. Teixeira, Ana L. T. O. Nascimento

**Affiliations:** 1 Laboratório de Desenvolvimento de Vacinas, Instituto Butantan, Avenida Vital Brasil, São Paulo, Brazil; 2 Programa de Pós-Graduação Interunidades em Biotecnologia, Instituto de Ciências Biomédicas, Universidade de São Paulo, São Paulo, Brazil

**Keywords:** *Leptospira*, leptospirosis, recombinant protein, inflammatory response, mammals cell lines, immune evasion

## Abstract

Pathogenic *Leptospira* spp. are the causative agents of leptospirosis, a significant zoonotic disease that has emerged as a crucial public health concern. This study aims to evaluate the interactions of two *L. interrogans* proteins, LIC_10499 and LIC_12339, with host components as well as with endothelial and epithelial cells. The coding sequences (CDSs) for LIC_10499 and LIC_12339 were cloned, expressed in *Escherichia coli*, and successfully purified from inclusion bodies. Both recombinant proteins demonstrated interactions with fibronectin, fibrinogen, and plasminogen (PLG). Notably, these proteins were capable of sequestering PLG from normal human serum (NHS). In the presence of an activator, the bound PLG is converted to plasmin (PLA), a broad-spectrum protease involved in pathogen invasion and immune evasion. Additionally, LIC_10499 and LIC_12339 were found to bind to complement system regulators, including factor H and C4b-binding protein, as well as to components C7, C8, and C9. We observed that the formation of C9 complexes was inhibited in the presence of recombinant proteins, and a higher survival rate of *E. coli* was noted when the proteins were incubated with NHS. The protein rLIC_10499 was able to bind to both monolayer and suspension cells of HMEC, Ea.hy926, and HEK293T, whereas rLIC_12339 only bound to HEK293T suspension cells. A significant production of IFN-γ was detected after 24 h when HEK293T epithelial cells were incubated with rLIC_10499, while a modest production of IL-6 and IL-8 was observed. No cytokine production occurred when HEK293T cells were stimulated with rLIC_12339. Collectively, these findings suggest that these proteins play a role in leptospiral immune evasion and have the potential to induce an inflammatory response in host cell monolayers.

## Introduction

Leptospirosis is a prevalent zoonotic disease caused by pathogenic species within the genus *Leptospira*. It is estimated that there are approximately 1.03 million severe cases and 58,900 deaths globally each year (1). Recently, the impact of global climate change, which has led to increased heavy rainfall and flooding, has made leptospirosis a significant public health challenge. In severe cases, such as Weil’s disease or leptospiral pulmonary hemorrhagic syndrome, fatality rates can exceed 50%, particularly in resource-limited settings (2–4). Leptospirosis also poses a considerable threat to livestock, resulting in issues such as abortion, stillbirth, reproductive failure, and milk drop syndrome (5). While vaccination of animals is an effective strategy to prevent the disease, renal colonization often persists, allowing these animals to become carriers of *Leptospira* and continue shedding viable bacteria into the environment (6, 7). Furthermore, the existing vaccines provide only short-term protection and are based on lipopolysaccharides, limiting their efficacy to the specific leptospiral serovars included in the vaccine formulation (2). Therefore, a comprehensive understanding of host-pathogen interactions is crucial for identifying virulence factors and for the development of a more effective vaccine.

Pathogenic *Leptospira* species disseminate throughout the bloodstream, colonizing vital organs such as the kidneys, liver, and lungs. This colonization results in endothelial disruption, leading to hemorrhage and organ dysfunction. One of the first humoral defenses that invading bacteria encounter is the complement system. This system operates through classical, lectin, and alternative pathways, all converging on the hydrolysis of C3 and the formation of the membrane-attack complex (MAC). The MAC creates pores in the cell membranes of invading pathogens, ultimately leading to their death (8). However, pathogenic *Leptospira* have developed mechanisms to evade complement-mediated killing (9). They possess a diverse array of surface proteins that hinder the activation of the complement system. Notably, multiple adhesins, such as LigA, LigB, and members of the Lsa family, interact with negative regulators like factor H (FH) and C4b-binding protein (C4BP). This interaction preserves their cofactor activity, facilitating factor I-mediated cleavage of C3b and C4b, thereby allowing the bacteria to survive in the host (10–12).

The capacity of pathogenic *Leptospira* to interact with epithelial and endothelial cells, as well as fibroblasts, significantly contributes to their dissemination (13). To date, only a few leptospiral proteins have been identified as ligands for cellular receptors. Monolayer and cell suspension assays have demonstrated selective binding of proteins such as OmpL1, LipL41, LipL46, and OmpL37 to both endothelial and epithelial cells. Recently, a novel protein designated as LIC_13355 has also been found to bind to endothelial and epithelial cell lines (14–16). In this study, we aim to investigate the functional roles of two *L. interrogans* proteins, LIC_10499 and LIC_12339. Both proteins contain in their sequences a domain of unknown function (DUF), which is present in leptospiral proteins with putative role in virulence (17, 18). Thus, we analyzed their capacity to bind host immune system proteins and assess their adhesion to endothelial and epithelial cells. Additionally, we evaluated the cytokine profile secreted following the incubation of these cells with both recombinant proteins. These findings are anticipated to bridge existing knowledge gaps in leptospiral pathogenesis and enhance strategies for leptospirosis control.

## Materials and methods

### Purified components

Bovine serum albumin (BSA), fetuin, collagen IV, collagen I, elastin, E-cadherin, plasma and cellular fibronectin, laminin, plasminogen (PLG), vitronectin, fibrinogen, heparin, heparan sulfate, chondroitin sulfate, chondroitin 4-sulfate and chondroitin sulfate B were acquired from Sigma-Aldrich (St. Louis, MO, USA). Integrins αVβ8, αMβ2, α5β1, αVβ5, αVβ1, αVβ3, αVβ6, α2bβ3 αLβ2 were purchased from R&D Systems (Minneapolis, MN, USA). Integrin α8 subunit was purchased from Abnova (Taoyuan, Taiwan). Factor H, C3b, C4b, C4-binding protein (C4BP), C7, C8 and C9 were purchased from Complement Technology (Tyler, TX, USA).

### Bacteria and human cell lines

*L. interrogans* serovar Copenhageni strains FIOCRUZ L1–130 and culture-attenuated M20 were grown in EMJH medium (Difco™, BD, Franklin Lakes, NJ, USA) supplemented with 10% *Leptospira* Enrichment EMJH (Difco™, BD). The attenuated-strain was used for genomic DNA isolation while the virulent FIOCRUZ L1–130 was used in cellular localization of the coding sequences*. E. coli* DH5α was used as cloning host, and *E. coli* BL21(DE3) and Shuffle T7 were used for recombinant protein expression; bacterial cells were grown in Lysogeny Broth (LB; Difco™, BD).

Human cell lines were purchased from ATCC (Manassas, VA, USA). The epithelial cell line HEK-293T (ATCC CRL-3216) and the endothelial cell line EA.hy926 (ATCC CRL-2922) were grown in Dulbecco’s Modified Eagle Medium (DMEM, D7777; Sigma-Aldrich) supplemented with 5% fetal bovine serum (FBS; Bionutrientes, São Paulo, SP, Brazil), 2 mM glutamine, and 100 U/mL penicillin-streptomycin (Lonza, Walkersville, MD, USA). HMEC-1 endothelial cells (ATCC CRL-3243) were grown in MDCB 131 (M8537; Sigma‐Aldrich) medium supplemented with 10% FBS, 10 ng/mL epidermal growth factor (E9644; Sigma‐Aldrich), 1 μg/mL hydrocortisone (H0888; Sigma‐Aldrich), and 10 mm glutamine (ATCC). Cells were cultured at 37°C under 5% CO2 atmosphere and tested for mycoplasma contamination before the assays were performed.

### *In silico* analysis

CELLO (19) (https://cello.life.nctu.edu.tw) and SignalP (20) (https://services.healthtech.dtu.dk/services/SignalP-6.0/) programs were used to detect signal peptides and cellular localization of the coding sequences LIC_10499 and LIC_12339. Localization was also analyzed using PSORT (21) (https://psort.org/psortb/) and SOSUI (22) (https://harrier.nagahama-i-bio.ac.jp/sosui/sosuigramn/sosuigramn_submit.html) programs. For the study of domains and regions of interest, InterPro (23) (https://www.ebi.ac.uk/interpro/), and SMART (24) (https://smart.embl.de/) programs were used. The tertiary structure of the protein was modeled using the AlphaFold Server (25) (https://alphafoldserver.com/welcome) program. The isoelectric point and molecular mass of the protein were calculated using the ProtParam (26) (https://web.expasy.org/protparam/) program.

### Triton X-114 fractionation

Log-phase cultures of *L. interrogans* serovar Copenhageni strain Fiocruz L1–130 were centrifuged at 2500 × g for 30 min at 4°C, washed three times with PBS, resuspended in Triton X-114 extraction buffer (10 mM Tris-HCl pH 8.0, 1% Triton X-114, 150 mM NaCl) and incubated overnight at 4°C. The mixture was again centrifuged at 15,000 × g for 30 min at 4°C. Triton X-114 (2% of the volume) was added to the supernatant and the mixture incubated at 37°C for 1 h. Phases were separated by centrifugation (2000 × g, 5 min). The upper phase was labeled aqueous, containing cytoplasmic proteins, and the lower phase labeled detergent, comprising outer membrane proteins. The pellet was resuspended in urea buffer (10 mM Tris-HCl pH 8.0, 8 M Urea, 4 mM DTT and 1% SDS), vortexed for 5 min and centrifuged at 15,000 × g for 30 min at 4°C. The resulting supernatant was labeled pellet fraction, which contains the inner membrane proteins. Fractions were quantified by Bradford (Sigma-Aldrich), and similar quantities of protein were precipitated by the addition of ice-cold acetone, incubated for at least for 16h at –20°C. The precipitate was then centrifuged at 15,000 × g for 15 min, all acetone removed, and the pellet resuspended in 50 µL of SDS-PAGE running buffer for western-blotting analysis.

### Fluorescence-activated cell sorting for protein detection

Low passage *L. interrogans* serovar Copenhageni strain Fiocruz L1–130 cultures were centrifuged (2,000 × g for 15 min), washed in PBS and resuspended in PBS supplemented with 5 mM MgCl_2_. Mouse polyclonal sera against rLIC_10499, rLIC_12339, and rLipL46 (outer membrane control), as well as rVapB (cytoplasmic control) (1:50, v/v), were generated by immunization of BALB/c mice, as described below. Each serum, including the control pre-immune serum (1:50, v/v), was individually added to cell cultures and incubated overnight at 30°C. After washing, secondary FITC-conjugated anti-mouse IgG (1:100, v/v; Sigma-Aldrich) was added, and the cells were incubated for 2 h at 30°C. Cells incubated with medium only and those treated solely with the secondary antibody FITC-conjugated were used to assess instrument settings and background fluorescence, respectively. Cells were then washed and resuspended in PBS containing 2% paraformaldehyde. Fluorescence measurements were performed using a BD FACSCanto II flow cytometer. The resulting data are presented as histograms, in which the X-axis represents the relative fluorescence intensity of labeled cells and the Y-axis represents the corresponding cell counts. The proportion of FITC-positive cells was determined relative to the total cell population.

### Cloning, expression and purification of recombinant proteins

Selected genes were amplified, without the signal peptide sequence, from *L. interrogans* M20 genomic DNA. Leptospiral DNA extraction was made as described before. For LIC_10499, the following set of primers were used: 5’-ACTGGGATCCGATGCTCCTCCGAC-3’ (*Bam*HI) and 5’-ATCGCCATGGTTACGGTCCCGCAA-3’ (*Nco*I). For LIC_12339, designed set of primers were: 5’-AGCTGGTACCCATGGATCATCAAAAGCTAATTATTCGATCGCT-3’ (*Nco*I) and 5’-CCGGATCAAGCTTCGTTACTGCAGAATACATCTACCTCCC-3’ (*Hin*dIII). Restriction sites were added for cloning in pAE expression vector (27), shown in underscore. Amplicons were purified with Illustra GFx PCR DNA and Gel Band Purification kit (GE Healthcare, Chicago, IL, USA) and cloned into pAE plasmid. Successful cloning was confirmed by sequencing in automated ABI sequencer (PE Applied Biosystems, Foster City, CA, USA) using T7 (5′‐TAATACGACTCACTATAGGG‐3′) and pAER (5′‐CAGCAGCCAACTCAGTTCCT‐3′) primers.

Recombinant LIC_10499 expression was induced at 37°C for 3 h with 1 mM isopropyl-β-D-1-thiogalactopyranoside (IPTG, Sigma-Aldrich) in *E. coli* BL21(DE3). Expression of recombinant LIC_12339 was induced in *E. coli* Shuffle T7 at 18°C for 24 h with 1 mM IPTG. After protein expression, the culture was collected and lysed by high pressure in a PandaPlus 2000 homogenizer (GEA Niro Soavi, Parma, Italy). Fractions were separated by centrifugation (7,000 × g, 10 min). Protein purification was performed by washing inclusion bodies by sonication in washing buffer (50 mM TrisHCl pH 8, 200 mM NaCl, 1% Triton X-100, and 1 M Urea), followed by solubilization in denaturing buffer. Purified protein was refolded by dialysis in PBS 1x pH 7.4 buffer at 4°C, where urea concentrations were reduced to zero.

### Secondary structure of recombinant proteins by circular dichroism

The recombinant proteins were dialyzed in 1 liter of sodium-phosphate buffer (0.5 M Na_2_HPO_4_; 1M NaH_2_PO_4_) at 4°C, three times at 1-h intervals. CD was measured using a cuvette with a 1 mm optical path at intervals of 0.5 nm/s and captured on a Jasco J-810 model spectropolarimeter (Japan Spectroscopic, Japan). The expressed spectra were measured in terms of residual molar ellipticity (Θ × L × C × 10 ^3^), where Θ (degrees) is the ellipticity, L (cm ^2^) refers to the optical path length, and C (dmol −1) is the concentration of the protein. The average readings were used for the analysis of secondary structures by submitting them to BeStSel software (28) (https://bestsel.elte.hu/index.php).

### Immunization and splenocyte proliferation assay

Recombinant protein suspension (10 µg in 250 µL of PBS 1x pH 7.4 + 4 mM Al(OH)_3_) was inoculated 3 times into female BALB/c mice at approximately 15-day intervals. Blood was collected via retro-orbital plexus 15 days after each dose. As a control, one group was immunized with PBS 1x pH 7.4 + 4 mM Al(OH)_3_. Produced sera were treated according to Gruber and Zingales (29). After immunization, mice were sacrificed and their spleen aseptically removed. Spleens were macerated in RPMI 1640 containing 2 mM L-glutamine, 100 IU/mL of penicillin and 100 µg/mL of streptomycin. After erythrocyte lysis, splenocytes were centrifuged (180 × g, 10 min), medium discarded and cells resuspended in 1 mL of RPMI supplemented with 10% FBS. Cell culture plates were seeded with 5x10^5^ cell/well and incubated overnight at 37°C with 5% CO_2_ atmosphere. Splenocytes were stimulated with 5 µg/mL of recombinants proteins and concanavalin A (ConA; Sigma-Aldrich) or purified *E. coli* LPS were used as a positive control. Medium alone was used as negative control. Cells were cultured for 48h at 37°C with 5% CO_2_ atmosphere, and proliferation rate was determined by the incorporation of bromodeoxyuridine (BrdU) in DNA synthesis, using BrdU ELISA colorimetric kit (Roche Diagnostic, Indianapolis, IN, USA), following the manufacturer’s protocol.

### Protein reactivity to MAT+ human samples

Leptospirosis human serum samples, as confirmed by microscopic agglutination test (MAT+), were obtained from Fundação Oswaldo Cruz (FIOCRUZ, Rio de Janeiro, Brazil). NHS (Sigma-Aldrich) was used as a negative control to assess assay specificity and for cutoff determination. Recombinant proteins were diluted in PBS to a final concentration of 250 ng/well and used to coat 96-well microtiter plates overnight at 4°C. Following incubation, plates were washed three times and blocked with PBS-T/1% BSA for 2 h at 37°C. After blocking, wells were washed and MAT-positive serum samples, diluted 1:200 in PBS, were added to each well. Plates were incubated for 1 hour at 37°C. Subsequently, wells were washed three times and horseradish peroxidase (HRP)-conjugated anti-human IgG antibody (1:5,000 v/v; Sigma-Aldrich) was added with further 1 h incubation at 37°C. Plates were washed and the reaction was developed by adding citrate-phosphate buffer pH 5 with 1 mg/mL o-phenylenediamine dihydrochloride (OPD; Sigma-Aldrich) and 1 µL/mL H_2_O_2_. Reactions were stopped by adding 4 N H_2_SO_4_. Readings were taken at 490 nm. To establish the cutoff value, NHS samples were processed in parallel with tested samples. Cutoff was defined as the mean absorbance value of the NHS control samples plus three times the standard deviation within control samples (mean + 3 SD). Samples with absorbance above the cutoff value are considered positive.

### Purified components binding screening and dose-response curves

The interaction of recombinant proteins was tested by ELISA in 96-well plates (Costar^®^ High Binding REF 3590; Corning Incorporated, Kennebunk, ME, USA).Extracellular matrix (ECM), plasma or complement system components (1 µg/well, except for vitronectin, 250 ng/well), glycosaminoglycans (GAGs), and integrins (100 µg/well) were adsorbed for 16 h at 4°C. Wells were washed and blocked with PBS-T/1% BSA for 2 h at 37°C. Plates were washed again and incubated with 1 µg of recombinant protein in PBS-T/1% BSA for 2 h at 37°C. Interaction was detected with anti-his antibodies conjugated with peroxidase (1: 10,000 v/v; Sigma-Aldrich) diluted in PBS-T/1% BSA for 1 h at 37°C. After washing, reactions were developed as described above; BSA and fetuin were used as negative controls. Components that showed statistical difference were tested in a dose-response curve. Similar to binding assay, ELISA plates were coated with each component diluted in PBS and incubated for 16 h at 4°C. Plates were washed three times, blocked with PBS-T/1% BSA for 2 h at 37°C, and washed three times. Recombinant protein was added to the first well of each row and serially diluted in a 1:1 proportion. The plate was incubated for 1 h at 37°C and washed three times. HRP-conjugated anti-His tag antibody (1:10,000 v/v; Sigma-Aldrich) was added and the plate incubated for 1 h at 37°C. The reaction was developed as described above.

### PLG uptaking from NHS

ELISA plates were coated with 1 µg of recombinant protein in PBS and incubated for 16 h at 4°C. Wells were washed and blocked with PBS-T/1% BSA for 2 h at 37°C. After washing, NHS (Sigma-Aldrich) diluted in PBS-T/1% BSA (20, 15, 20 and 5% v/v) was added to wells and incubated for 1 h at 37°C. After washing, polyclonal anti-PLG was added (1:5,000 v/v) and incubated for 1 h at 37°C. Wells were washed and HRP-conjugated anti-mouse IgG was added (1:10,000 v/v; Sigma-Aldrich) and incubated for 1 h at 37°C. The plate was washed six times and reaction developed as described above.

### Binding to PLG’s kringle domain assay

ELISA plates were coated with 1 µg/well of recombinant protein or BSA, used as a negative control, in PBS for 16 h at 4°C. Plates were washed, blocked with PBS-T/1% BSA at 37°C for 2 h and was added 1 µg/well of purified PLG with 0, 2 or 20 mM aminocaproic acid (ACA), incubating the reaction for 2 h at 37°C. After washing, bound PLG was detected with polyclonal anti-PLG antibody (1:5,000 v/v), followed by the addition of HRP-conjugated anti-mouse IgG antibody (Sigma-Aldrich, 1:10,000 v/v). Reaction was developed as described before.

### Plasminogen conversion assay

PLG conversion to plasmin (PLA) was assessed by the conversion of the chromogenic substrate D-Val-Leu-Lys 4-nitroanilide dihydrochloride (S) in the presence of urokinase-type plasminogen activator (uPa; Sigma-Aldrich). A 96 well plate was coated for 16 h at 4°C with 1 µg/well of each recombinant protein. As a negative control, BSA was used. After blocking with PBST-BSA 1%, 1 µg of purified PLG or 20% NHS in PBST/BSA was added to each well and incubated at 37°C for 2 h. Plates were washed and 5 ng of human uPA and 0.8 mM of PLA-specific substrate was added to each well. As control, each component of the reaction was omitted. The reaction was developed overnight at 37°C and absorbance was taken at 405 nm.

### Inhibition of zinc induced C9 polymerization

Purified C9 component was incubated at 37°C for 40 min with 1.25, 2.50 and 5.00 µg of recombinant protein in TBS buffer (Tris-HCl 50 mM pH 7.4, NaCl 200 mM). As a positive and negative control of the inhibition, 5 µg of rLIC_13259 (30) and fetuin was used. To each mixture, ZnCl_2_ was added to 50 nM and incubation proceeded for 2 h at 37°C. As a control of the polymerization, free C9 was incubated with or without the addition of ZnCl_2_. Each reaction was analyzed in native SDS-PAGE gradient gel.

### NHS susceptibility assay

NHS (Complement Technology, Tyler, TX, USA) was diluted to 1% in DPBS++ (DPBS pH 7.4, MgCl_2_ 1,5 mM, CaCl_2_ 0,5 mM) and incubated with or without 1 µM of recombinant protein at 37°C for 15 min. Exponential phase *E. coli* DH5α were collected, washed with DPBS++ and concentration adjusted to 2.10^4^ cells/mL. A total 100 µL of NHS diluted was added to 100 µL of *E. coli* suspension, and the mixture incubated at 37°C for 1 h. Next, cells were placed in ice for 1 min and plated on LB-agar plates, which were incubated at 37°C for 16 h, and colony forming units were counted. As a negative control, cells were treated with PBS only or heat inactivated NHS (iNHS). The action of specific anti-LIC antibodies was assessed by incubating 1 µM of recombinant protein with its corresponding hyperimmune serum for 15 min at 37°C prior to incubation with NHS.

### Human cell line binding assay

Binding of recombinant protein to human cell line HEK-293T, EA.hy926 and HMEC-1 monolayers was assessed by ELISA and Western blotting. 96-well culture cell plates (Falcon 353916; Corning Incorporated) were seeded with 10^5^ cells and incubated overnight at 37°C in 5% CO_2_ atmosphere. Wells were washed with PBS-T and, to each well, 1 µg of each recombinant protein in DMEM supplemented with 1 µM aprotinin was added. Only DMEM + 1 µM aprotinin was added to control wells. The plate was incubated for 2 h at 37°C in 5% CO_2_ atmosphere with subsequent washing with PBS-T. The binding was fixed with paraformaldehyde 2% in PBS pH 7.4 for 30 min at RT. After washing, the plate was incubated with glycine 2% in PBS pH 7.4. HRP conjugated anti-His antibody (1:10,000) was added, followed by incubation for 1 h at 37°C. Binding was developed as described above.

To access the binding to the cell surface, 2X10^5^ trypsinized cells were incubated with 5 µg of recombinant protein in 200 µL of DMEM + 1 µM aprotinin for 2 h at 37°C in 5% CO_2_ atmosphere. After incubation, cells were pelleted by centrifugation (180 × g, 10 min) and the supernatant reserved. The cell pellet was resuspended in 200 µL of DPBS. Fractions were boiled in SDS buffer and analyzed by western-blotting. In brief, after transferring proteins to the membrane, it was blocked overnight with 10% non-fat dried milk in PBS containing 0.05% of PBS-T. Next day, the membrane was probed with HRP-conjugated anti-His tag antibody (1:10,000 v/v; Sigma-Aldrich) for 1 hour at RT, washed 5 times with PBS-T and developed by an ECL prime Western Blotting detection (Cytiva, Global Life Sciences Solutions USA LLC, Marlborough, MA 01752, USA).

### Multiplex analysis

Cell culture 24-well plates were seeded with 2X10^5^ HEK-293T cells in 500 µL of DMEM medium and incubated overnight at 37°C under 5% CO_2_ atmosphere. To each well, 1 µM of recombinant protein in DMEM medium, supplemented with 1 µM aprotinin, was added. Cells were incubated at 37°C under 5% CO_2,_ and supernatant aliquots were collected at 2, 4, 6, 24, 48 and 72 h post-reaction. Cytokine multiplex analysis was made using MILLIPLEX^®^ Human Cytokine/Chemokine/Growth Factor Panel A kit (Sigma-Aldrich) in Milliplex Luminex (Luminex, Austin, TX, USA).

### Statistical analysis

Graphs, curves, and statistical analyses were performed using GraphPad Prism v6.05 software. For bindings, two tailed Student t-test was performed against negative controls. Dose-response curves and K*_D_* were calculated using “non-linear regression” options and considering saturation as “one site-specific binding”.

### Ethics statement

This study was performed according to the guidelines outlined by the Brazilian National Council for Control of Animal Experimentation (CONCEA), which follows international guidelines for animal welfare and the principles of the 3Rs. Experimental protocols comply with the ARRIVE guidelines and were approved by the Ethic Committee on Animal Use of the Butantan Institute, São Paulo, Brazil (protocol no. 2840200422). Mice were housed in a BSL1 animal facility, in micro isolators with individual ventilation and temperature and light cycle control. Animals received food and water *ad libitum* and manipulation was performed by trained personnel. Animals were euthanized by intraperitoneal injection of ketamine (300mg/kg) and xylazine (30mg/kg).

## Results

### *In-silico* analysis

The coding sequences LIC_10499 and LIC_12339 from *L. interrogans* serovar Copenhageni were analyzed *in silico* for their conservation, structural features and cellular localization. Signal peptide prediction using the SignalP 6.0 (20) web server indicates that LIC_10499 contain a type I signal peptide (SpI) with cleavage site between amino acids 23 and 24. In contrast, no signal peptide was identified for LIC_12339. The software tools SOSUI-GramN (22) and Cello (19) predicted that LIC_10499 is extracellular, while LIC_12339 is predicted to be periplasmic and extracellular, respectively.

**Figure 1:**
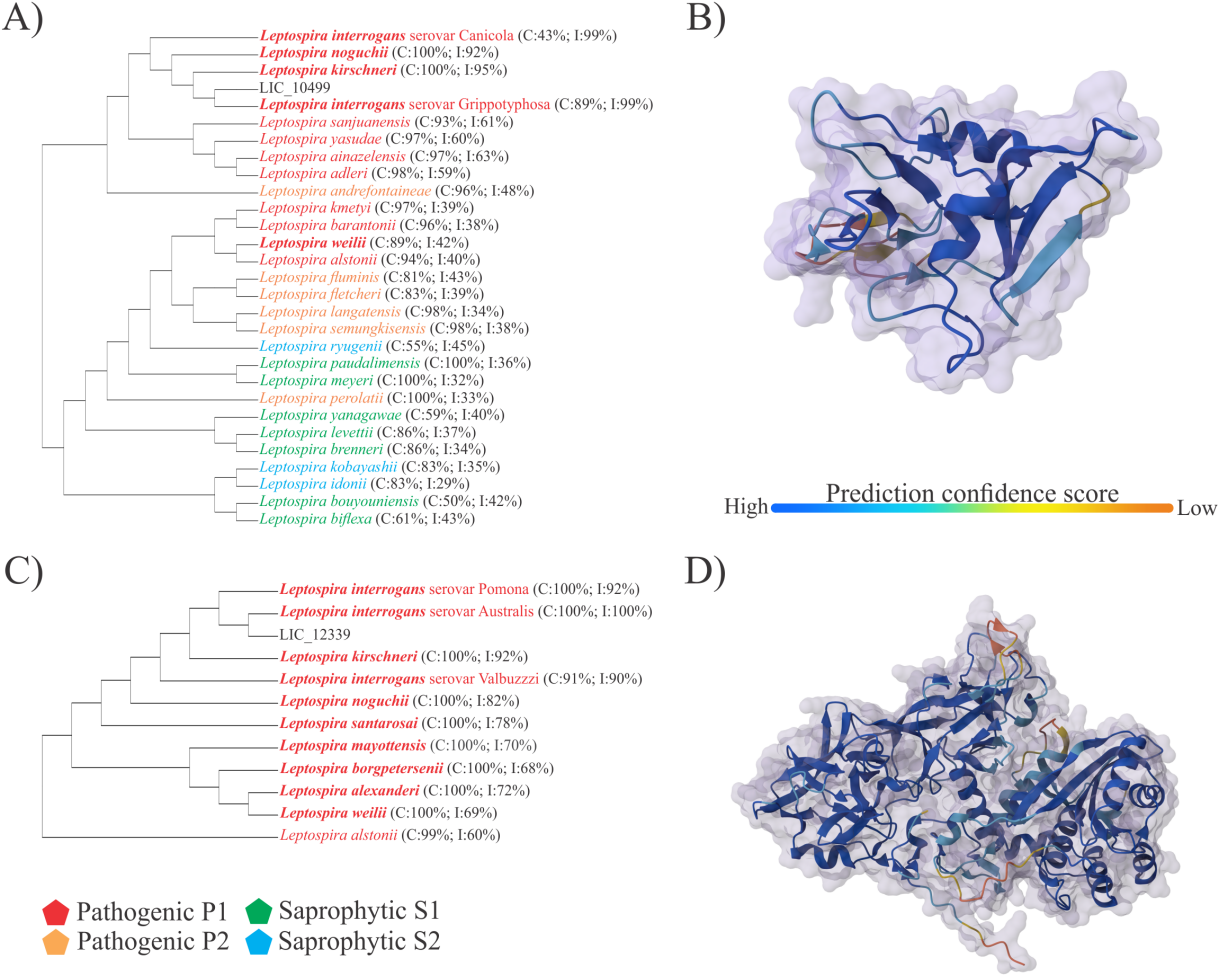
*In silico* analysis of LIC_10499 and LIC_12339. Conservation analysis of the LIC_10499 and LIC_12339 CDSs was performed using BLAST and tertiary protein prediction via NCBI and the AlphaFold Server web services. In the phylograms, pathogenic P1+ species are represented in bold, with conservation (C) and identity (I) values described. **(A)** The phylogram shows that LIC_10499 is conserved among Leptospira species, with conservation and identity values ranging from 43% to 100% and 38% to 99%, respectively; **(B)** The predicted tertiary structure of LIC_10499 is depicted, showing a high confidence score for most of the protein; **(C)** BLAST analysis of LIC_12339 indicates its presence only in pathogenic species; **(D)** The predicted model for LIC_12339 displays a protein structure with a high confidence score.

The SMART (22) web server analysis revealed that LIC_10499 includes a domain of unknown function (DUF1566). According to gene ontology (31, 32) (GO:0004197), this domain is associated with cysteine-type endopeptidase activity and contains a C-type lectin domain (PROSITE PDOC00537). Orthologous groups identified through eggNOG (33) suggest that this domain is clustered with FimH-like proteins (ENOG4105PV2). FimH proteins, found in the fimbriae of *E. coli*, serve as the adhesive component of type I pili and also act as a TLR4 ligand (34). BLAST (35) analysis of the LIC_10499 protein sequence indicates that this protein is present in various species of *Leptospira*, demonstrating high conservation among pathogenic P1+ species (**Figure 1A**).

The tertiary structure of LIC_10499, modeled using the AlphaFold Server (25) (**Figure 1B**), exhibits a high confidence score. A search of the RSCB PDB using this predicted model reveals similarities with proteins from other pathogens (36–43). Domain analysis of LIC_12339 indicates that this protein contains a DUF1561, which belongs to the protein family PF07598, recently designated as virulence-modifying proteins, and is found exclusively in pathogenic *Leptospira* species (44). BLAST analysis of LIC_12339 further confirms that this protein is restricted to pathogenic species of *Leptospira* (**Figure 1C**). Additionally, the modeled tertiary structure of LIC_12339 (**Figure 1D**) was utilized to search for similarly structured proteins in the RSCB PDB; however, no virulence-related proteins were identified.

### Cellular localization of the coding sequences LIC_10499 and LIC_12339

**Figure 2:**
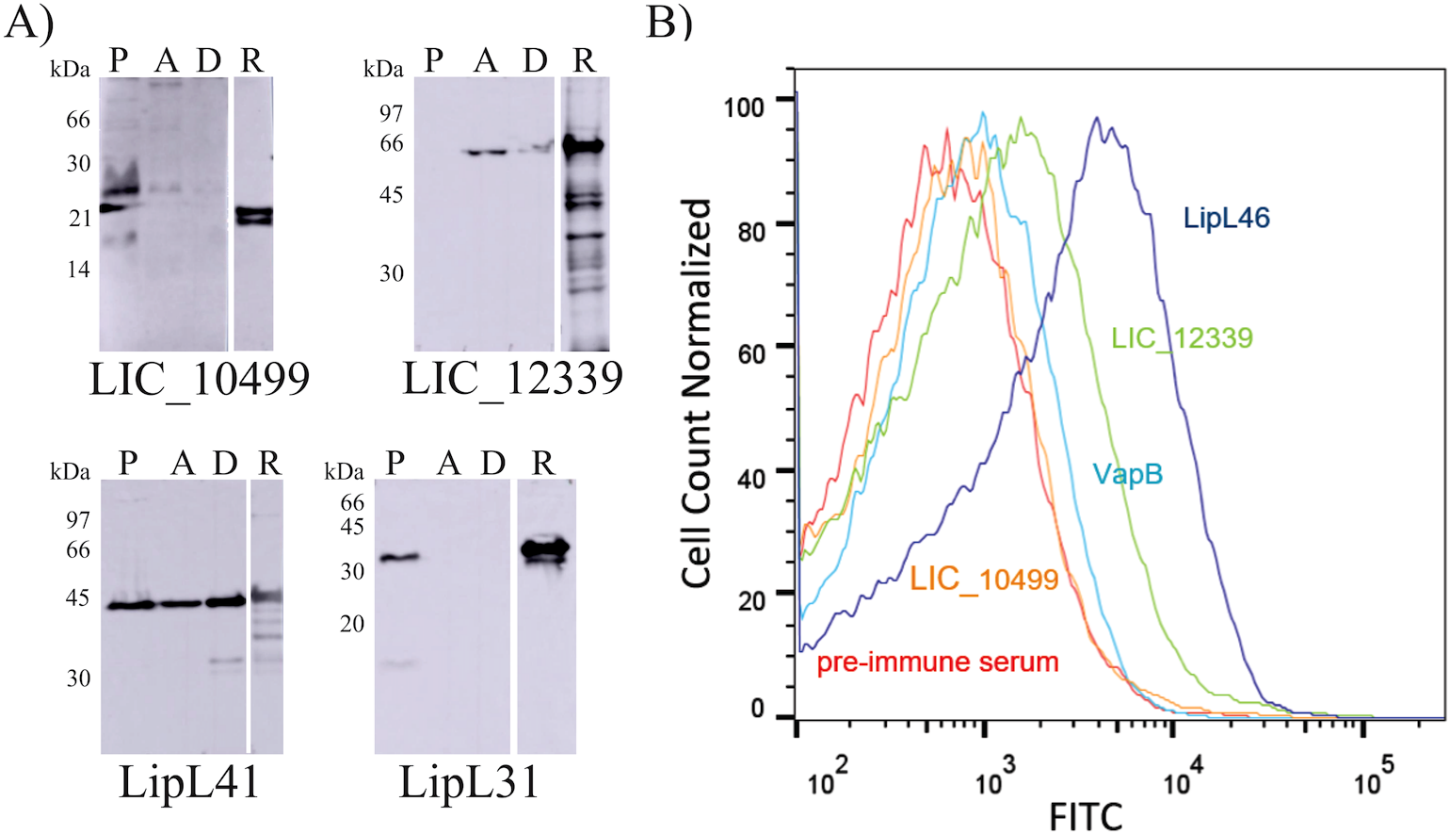
Subcellular localization of the coding sequences (CDSs) in L. interrogans serovar Copenhageni Fiocruz L1-130. **(A)** Western blot analysis of fractionated extracts: *L. interrogans* cultures at the logarithmic phase were collected, washed with PBS, and protein fractions were separated using Triton X-114 fractionation. Proteins in the cell extracts were detected using polyclonal antibodies (anti-rLIC_10499 1:800 v/v, anti-rLIC_12339 1:5,000 v/v, anti-LipL41 1:5,000 v/v, and anti-LipL31 1:5,000 v/v); detection was performed with HRP-conjugated anti-mouse IgG (1:5,000 v/v). LipL41 and LipL31 were used as controls for outer and inner membrane proteins, respectively. AF: aqueous fraction; P: pellet fraction; D: detergent fraction; R: recombinant protein. **(B)** FACS localization histogram: anti-LipL46 and anti-VapB antibodies were employed as external and internal protein controls, respectively. Also included is the histogram of cells incubated with pre-immune serum to assess fluorescence background.

The subcellular localization of proteins was assessed using Triton X-114 fractionation of *L. interrogans* strain L1–130 cells, allowing for the separation of outer membrane, inner membrane, and cytoplasmic proteins into detergent, pellet, and aqueous fractions, respectively. Western blot analysis with immune serum against the target protein revealed that LIC_10499 was present in both the pellet (P) and aqueous (AF) fractions, indicating its localization in the inner membrane of *Leptospira* cells (**Figure 2A**). These findings are consistent with previous proteomic analyses that demonstrated the presence of LIC_10499 in the cytoplasm, surface, and inner membrane fractions of *L. interrogans* serovar Copenhageni (45, 46). Conversely, LIC_12399 was detected in the aqueous (AF) and detergent (D) fractions, suggesting its localization at the outer membrane of *L. interrogans*. To validate the subcellular fractionation, the recombinant protein LipL41 was used as positive control for outer membrane protein (47) and LipL31 was included as control of inner membrane protein.

In addition to western blot results, a localization analysis was conducted using flow cytometry (**Figure 2B**). Intact leptospires were incubated with immune sera raised against the recombinant proteins, with anti-LipL46 and anti-VapB serving as controls for outer membrane and cytoplasmic proteins, respectively. Pre-immune serum was used as a control for nonspecific background labeling. In the histograms, a shift along the X-axis corresponds to increased FITC labeling among the total cell population. Approximately 63.7% of cells were labeled for LipL46, while 40.4% were labeled for VapB. Although VapB is a cytoplasmic protein and thus not expected to be surface-labeled, the observed fluorescence likely results from nonspecific binding combined with the background signal of 26.9% detected with the non-immune serum. Using the fluorescence intensities of LipL46 and VapB as reference parameters for protein localization, the labeling of 49.8% for LIC_12339 suggests an outer membrane localization, as its histogram profile resembles that of LipL46. In contrast, LIC_10499 appears to be internally localized, with 24.7% labeled cells, a value comparable to that of the non-immune serum, consistent with an inner membrane-associated protein.

### Recombinant protein expression, purification, and antiserum production

**Figure 3:**
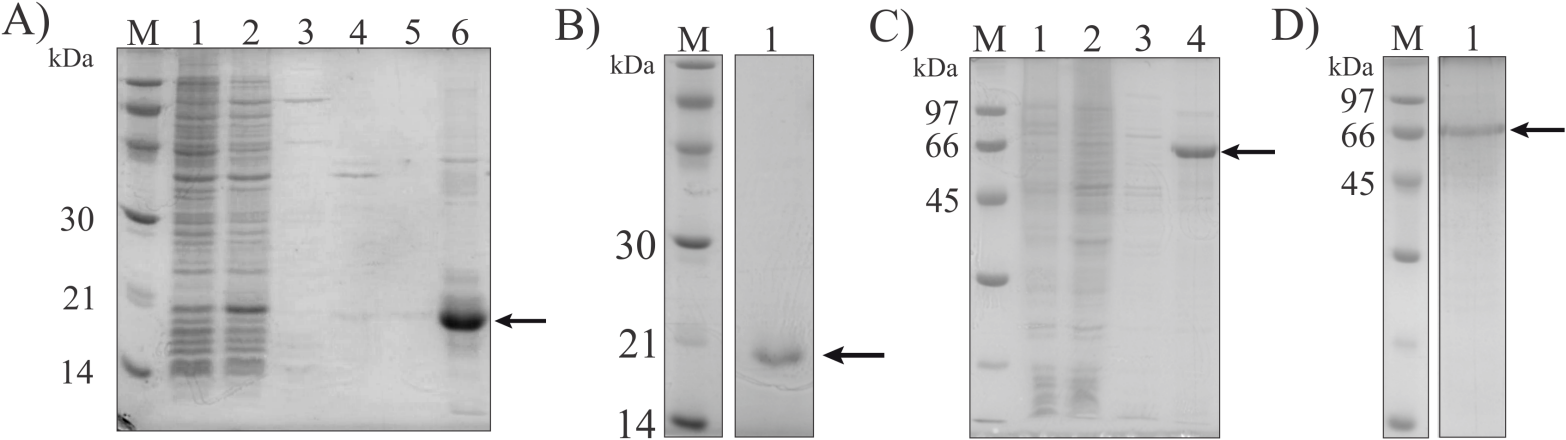
Analysis of recombinant protein expression and purification by SDS-PAGE. **(A)** rLIC_10499 production and purification analysis. 1: pre-induction extract, 2: post-induction extract, 3: soluble fraction, 4 and 5: urea washing fractions, 6: insoluble fraction. **(B)** refolded and dialyzed rLIC_10499. **(C)** Purification analysis of rLIC_12339. 1: pre-induction extract, 2: post-induction extract, 3: induction soluble fraction, 4: induction insoluble fraction. **(D)** refolded and dialyzed rLIC_12339. M: molecular mass protein marker.

Coding sequences LIC_10499 and LIC_12339 were PCR amplified from *L. interrogans* strain M20 and the respective generated fragments of 471 pb and 1842 pb were subsequently cloned into the pAE vector. The recombinant vectors underwent BLAST analysis, which revealed no mutations and 100% identity between the sequences in *L. interrogans* strains M20 and FIOCRUZ L1 130. Expression hosts *E. coli* BL21(DE3) and ShuffleT7 were transformed with pAE-LIC10499 and pAE-LIC12339, respectively, for heterologous protein expression. Cultures were induced with 1 mM IPTG, with LIC_10499 expressed at 37°C for 3 h and LIC_12339 at 18°C for 16 h. SDS-PAGE analysis showed that rLIC_10499 was expressed as inclusion bodies (**Figure 3A**, lane 6). Following purification and dialysis in PBS, the purity of rLIC_10499 was confirmed by SDS-PAGE, revealing a major protein band at the expected molecular mass of 16,9 kDa (**Figure 3B**, lane 1). Similarly, rLIC_12339 was detected in the insoluble fraction (**Figure 3C**, lane 4). After washing with urea and dialysis, rLIC_12339 exhibited a high degree of purity and expected molecular mass of 68,9 kDa (**Figure 3D**, lane 1). Secondary structure by CD spectral analysis was, unfortunately not possible to be performed, since both proteins were instable and precipitated in the applicable buffer.

Purified recombinant proteins were used to immunize mice three times at 15-day intervals, with immune serum collected 15 days after each immunization. Two weeks following the final dose, the mice were euthanized, and their spleens were removed for splenocyte proliferation assays. The antiserum titer against rLIC_10499 was measured as 25,600, yet no splenocyte proliferation was observed when exposed to 1 µg of rLIC_10499. In contrast, rLIC_12339 showed an antiserum titer exceeding 204,800, indicating immunogenic activity; however, no splenocyte proliferation was observed following rLIC_12339 exposure in the BRDU assay (data not shown). These results indicate that both proteins promote an exclusive humoral response, similar to other leptospiral recombinant proteins (48), Gaspar et al., unpublish results). Modulation of the immune response by a new adjuvant is probably necessary for the activation of an efficient adaptive immune response. Very low reactivity between both recombinant proteins and leptospirosis serum samples was detected, suggesting low expression level of the coding sequences during infection (data not shown).

### Recombinant proteins bind to host complement system proteins affecting its function

**Figure 4:**
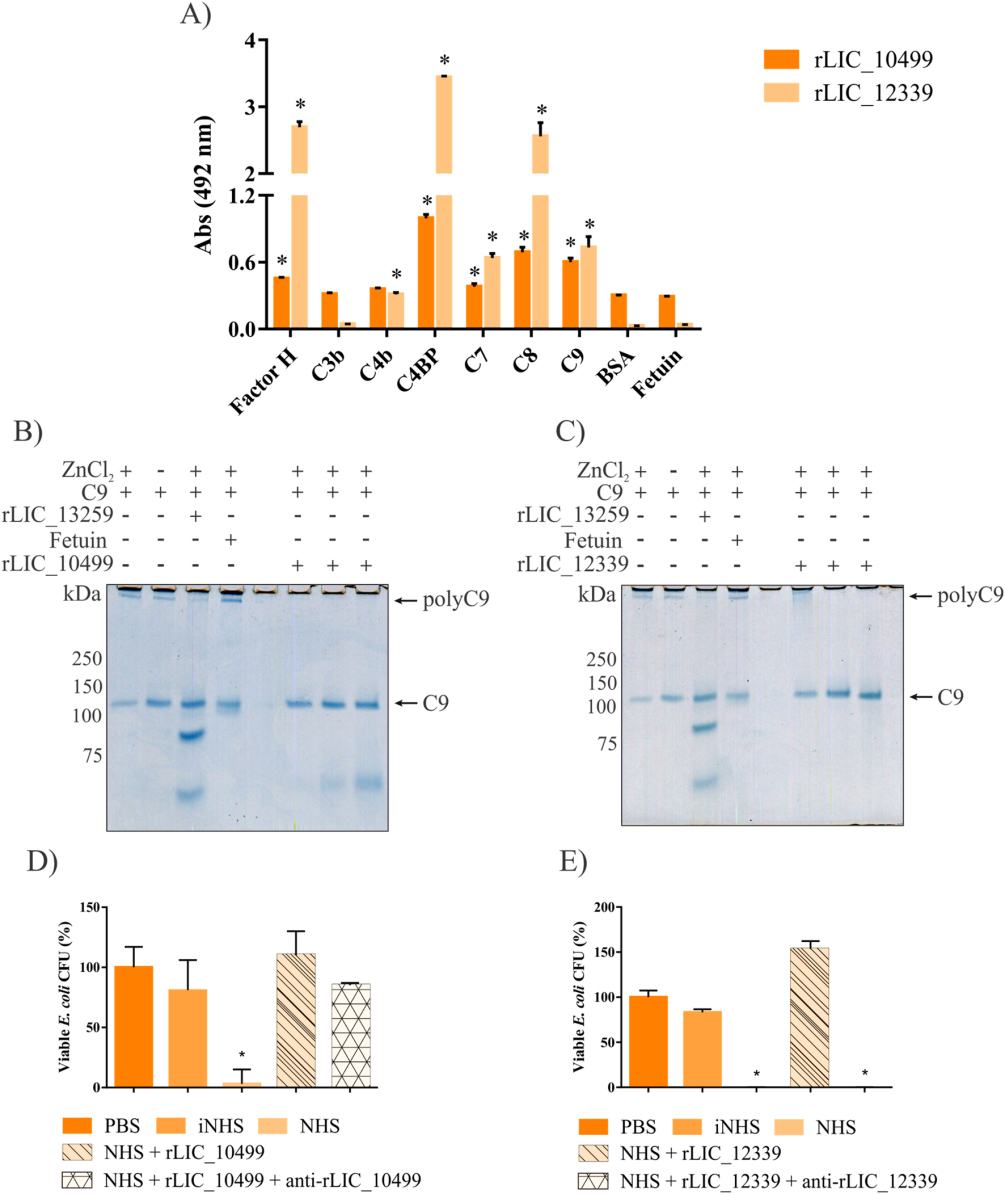
Interaction of rLIC_10499 and rLIC_12339 with complement system components. **(A)** ELISA plates were coated with 1 µg/well of each purified component. Subsequently, 1 µg/well of recombinant protein was added and allowed to interact for 2 h at 37°C. Bound proteins were detected using HRP-conjugated anti-His antibody (1:10,000 v/v). Binding specificity was tested against negative controls BSA and fetuin using Student’s t-test (*p < 0.05). **(B, C)** Native-PAGE analysis of C9 polymerization in the presence of recombinant proteins. In TBS buffer, 3 µg of C9 protein was incubated with 1.25, 2.5, and 5 µg of rLIC_10499 **(B)** and similarly with rLIC_12339 **(C)**. As positive and negative inhibition controls, 5 µg of rLIC_13259 and 5 µg of fetuin were used, respectively. ZnCl2 was added to a final concentration of 50 nM, followed by a 2-h incubation. C9 alone, with or without ZnCl2, was used as a polymerization control. Aliquots of each reaction were analyzed by native-PAGE and stained with Coomassie blue. **(D, E)** Increased survival of *E. coli* exposed to NHS in the presence of recombinant proteins. Live *E. coli* DH5α cells were treated with 0.5% NHS supplemented with 1 µM of rLIC_10499 **(D)** or rLIC_12339 **(E)** protein for 1 h. Cells were then seeded on agar plates and incubated overnight at 37°C. As negative controls, cells were treated with heat-inactivated NHS (iNHS) or PBS alone. To assess the effect of the proteins, antibodies against each protein (1:100 v/v) were used prior to the addition of NHS. CFU counts were converted to percentages, considering cells incubated with PBS as 100%.

Pathogenic *Leptospira* can evade complement-mediated killing by interacting with complement molecules, which impair complement activation. To investigate whether rLIC_10499 and rLIC_12339 could play a role in immune evasion via complement, their binding to purified complement system proteins from the host was assessed using ELISA. As a negative control, the binding of the recombinant proteins to BSA and fetuin was also evaluated. Recombinant LIC_10499 was found to bind to Factor H, C4BP, C7, C8, and C9 proteins, while rLIC_12339 bound to Factor H, C4b, C4BP, C7, C8, and C9 (**Figure 4A**). Binding specificity was tested using dose-response curves (**Supplementary Figure S1**), and dissociation constant (K*_D_*) was calculated from binding saturation curves. K*_D_* values are depicted in **Table 1**.

**Table 1:** Dissociation constants (K*_D_*) of the recombinant proteins binding to ECM, plasma and complement system components (μM).

Origin	Component	rLIC_10499	rLIC_12339
		K*_D_* (µM)	
ECM	Laminin	0.212 ± 0.027	–
	Cellular fibronectin	0.147 ± 0.020	-
	Type I Collagen	0.275 ± 0.031	–
	Type IV Collagen	0.303 ± 0.029	-
Plasma	PLG	0.049 ± 0.003	0.013 ± 0.002
	Fg	0.268 ± 0.021	0.144 ± 0.026
	Plasma fibronectin	0.276 ± 0.018	0.042 ± 0.05
Complement system	Factor H	0.134± 0.021	0.070 ± 0.009
	C4b	–	0.084 ± 0.032
	C4BP	0.019 ± 0.004	0.067 ± 0.009
	C7	0.046 ± 0.012	0.145 ± 0.063
	C8	0.035 ± 0.005	0.030 ± 0.03
	C9	0.401 ± 0.110	0.217 ± 0.022

The binding of proteins from pathogenic bacteria to C9 can inhibit its polymerization, thereby preventing cell lysis by obstructing the formation of the membrane attack complex (MAC). Tschopp (49) demonstrated that the addition of Zn²^+^ ions can accelerate C9 circular polymerization *in vitro*; this technique was utilized to assess the impact of various pathogen proteins on C9 oligomerization (9, 30, 50, 51). To evaluate whether the presence of both rLIC_10499 and rLIC_12339 could hinder circular C9 formation, these proteins were incubated with C9 in the presence of Zn²^+^, including the positive control rLIC_13259 (30). It was observed that rLIC_10499 completely inhibited C9 formation, even at the lowest concentration tested (1,25 µg) (**Figure 4B**). Though rLIC_12339 also blocked the formation of C9 cylinders (**Figure 4C**), a higher concentration was required to fully inhibit their formation. In addition to their interaction with C9, both rLIC_10499 and rLIC_12339 have demonstrated the ability to bind to negative regulators of the complement system, including Factor H and C4BP. This recruitment of complement regulators appears to play a crucial role in allowing *L. interrogans* to evade complement-mediated killing (52–54). To investigate whether these recombinant proteins can hinder complement activity through this mechanism, we conducted experiments using live *E. coli* DH5α cells. These cells were exposed to NHS both in the presence and absence of 1 µM of the recombinant proteins, and their survival rates were assessed by measuring colony formation on LB-agar plates. The results showed that treatment of the bacteria with 1 µM of rLIC_10499 or rLIC_12339 in conjunction with NHS resulted in survival rates comparable to those of untreated bacteria or those treated with heat-inactivated NHS, suggesting that both proteins can hinder complement activity (**Figures 4D, E**). However, when rLIC_10499 was pre-treated with its corresponding immune serum, only partial activation of complement activity was observed, suggesting that the regions involved in the interaction of complement molecules are different from immunogenic epitopes. In stark contrast, incubation of NHS with 1 µM of rLIC_12339 alongside its immune serum resulted in complete activation of the complement system (**Figure 4E**). This finding highlights the significant role of rLIC_12339 in modulating complement activity.

### Recombinant proteins bind to host plasma components

**Figure 5:**
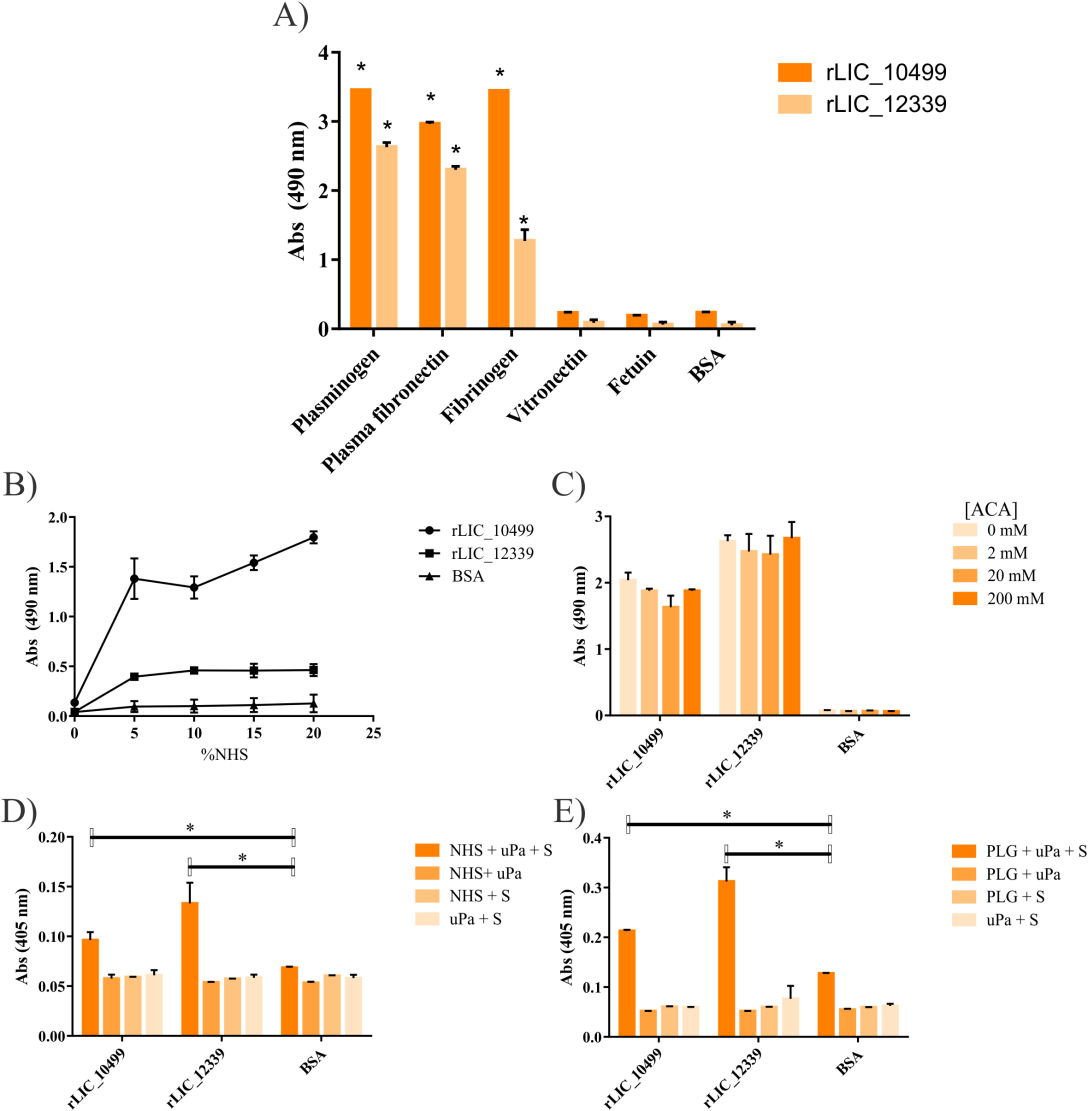
Interaction of recombinant proteins to human plasma components. In **(A)** binding of rLIC_10499 and rLIC_12339 to purified human plasma components. ELISA plates were coated with 1 µg/mL purified plasma proteins, except for vitronectin (250 ng/well). To wells, 1 µg/well of rLIC_10499 or rLIC_12339 was added and the plates incubated for 2 h at 37°C. Bound recombinant protein was detected using HRP conjugated anti-His antibody (1:10,000 v/v). Binding significance was assessed by Student’s t-test against negative controls BSA and Fetuin (*=p<0.05). **(B)** PLG capture from NHS: increasing concentrations of NHS diluted in PBS was added to ELISA plates coated with 1 µg/well of recombinant protein or BSA and left to interact for 2 h at 37°C. Bound PLG was detected using anti-PLG polyclonal antibody (1:10,000 v/v), followed by HRP conjugated anti-mouse IgG (1:5,000 v/v). **(C)** Competition of recombinant proteins binding to PLG in the presence of amino caproic acid (ACA). ELISA plates were coated with 1 µg of purified PLG. After blocking, 1 µg of recombinant protein with 0, 2, 20 or 200 µM ACA was added and incubated for 2 h at 37°C. Wells were washed and HRP conjugated anti-his antibody added (1:10.000). Binding of treated recombinant protein was compared to that with no ACA addition. **(D)** NHS (20% v/v) was added to wells in ELISA plates coated with 1 µg/well of recombinant protein or BSA, used as a negative control. Bound PLG conversion was assessed by the addition of 5 ng uPa + 0.8 mM of PLA-substrate (S). As a control, each component was omitted. **(E)** Likewise, purified PLG (1 µg/well) conversion in the presence of uPA and PLA-substrate. Bound PLG conversion was compared to BSA/PLG conversion by Student’s two-tailed t-test (*=p<0.05).

*L. interrogans*, similar to other pathogenic organisms, utilizes various strategies to evade the immune defenses of the host, thereby facilitating their spread. One such mechanism involves the acquisition of PLG and its subsequent conversion into PLA (55–58). Additionally, the binding of *L. interrogans* to fibrinogen serves as another tactic to prevent the formation of fibrin clots, further aiding in their dissemination (56, 59–63). In this study, we demonstrated that both rLIC_10499 and rLIC_12339 possess the ability to bind to PLG, plasma fibronectin, and fibrinogen in a dose-dependent manner. No binding was observed between the recombinant proteins and the negative controls BSA and fetuin (**Figure 5A**; **Supplementary Figure S2**). Notably, both recombinant proteins were capable of sequestering PLG from NHS (**Figure 5B**), and this interaction was shown to occur independently of the Kringle domain of PLG (**Figure 5C**). Furthermore, when rLIC_10499 and rLIC_12339 were bound to PLG, they facilitated its conversion into PLA in the presence of the PLG activator, uPA (**Figures 5D, E**). This observation suggests that these proteins exhibit significant proteolytic potential.

### LIC_10499 binds to ECM components and human cell lines *in-vitro*

**Figure 6:**
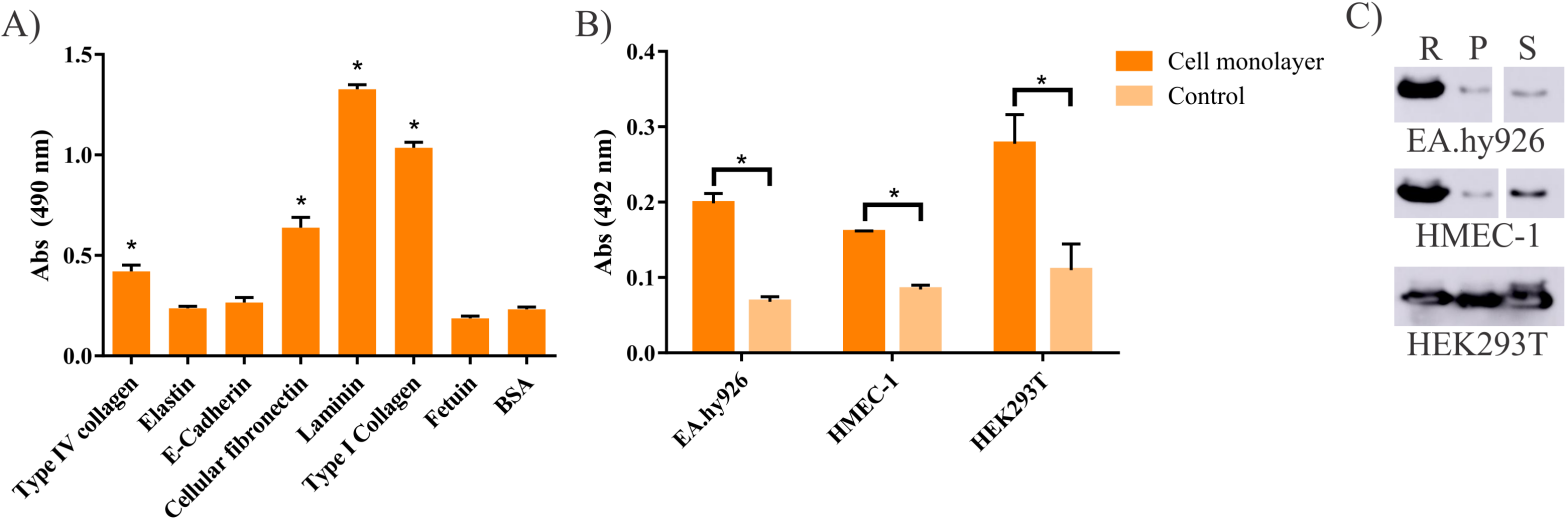
Interaction of rLIC_10499 with extracellular matrix components and human cells. **(A)** 96-well plates were coated with 1 µg/well of each purified component. To them, 1 µg/well of recombinant protein was added and the interaction incubated for 2 h. Bound rLIC_10499 was detected using anti-His antibody (1:10,000). Bound protein was compared to negative controls BSA and Fetuin by Student’s two-tailed t-test (*p=0.05). **(B)** Assessment of rLIC_10499 interaction to cell monolayers by ELISA and Western Blotting. Cell culture plates were seeded with 10^5^ cells/well and incubated overnight at 37°C with 5% CO_2_ atmosphere. Then, 1 µg of recombinant protein in DMEM supplemented with 1 µM aprotinin was incubated for 2 h at 37°C with 5% CO_2_. For the negative control, only DMEM + aprotinin were used. The interaction was fixed using 2% paraformaldehyde in PBS (pH 7.4) at room temperature, and then incubated with glycine 2% at room temperature. Protein adhesion was detected using HRP conjugated anti-His (1:10,000) antibody. Binding to cells was compared to those incubated with DMEM alone by Student’s two-tailed t-test (*p<0,05) **(C)** Binding of rLIC_10499 to cells in suspension. Recombinant protein (5 µg) was added to trypsinized cells (2.10^5^) in DMEM supplemented with 1 µM aprotinin, and left to interact for 2h at 37°C with 5% CO_2_ atmosphere. Cells were pelleted and the supernatant isolated and the cell pellet resuspended in PBS. Both fractions were boiled in SDS-running buffer. Fraction analysis was made by western-blotting using anti-His (1:10,000). R: recombinant protein, P: Cell pellet fraction, S: Supernatant fraction.

Several proteins from *L. interrogans* appear to play significant roles in the adhesion process to host cells, and to components of the ECM, thereby mediating crucial host-pathogen interactions (11, 13, 17, 64–66). To assess whether the recombinant proteins rLIC_12339 and rLIC_10499 interact with ECM components and mammalian cells, we conducted an interaction assay utilizing ELISA. This evaluation was performed using both adhered cells in monolayer cultures and cell suspensions. As shown in **Figure 6A**, rLIC_10499 was able to bind to collagen types I and IV, cellular fibronectin, and laminin. However, the interaction of both rLIC_12339 and rLIC_10499 with integrins and glycosaminoglycans (GAGs) was not detected. Moreover, no binding of rLIC_12339 to ECM components was observed (data not shown). The adhesion properties of rLIC_10499 to various cell lines, including endothelial cells (Ea.hy926 and HMEC-1) and epithelial cells (HEK293T), are illustrated in **Figure 6B**. Notably, rLIC_10499 demonstrated the ability to interact with all tested cell lines under both conditions—monolayer and suspension. The interactions observed with suspension cells suggest that rLIC_10499 may engage with specific cell surface receptors (**Figure 6C**). In contrast, rLIC_12339 was tested exclusively with the epithelial cell line HEK293T, and an interaction was identified only in the context of suspension cells. This finding aligns with the previously noted absence of binding to ECM components (data not shown).

### Cytokine production by HEK239T cell line after stimulation with rLIC_10499 and rLIC_12339

**Figure 7:**
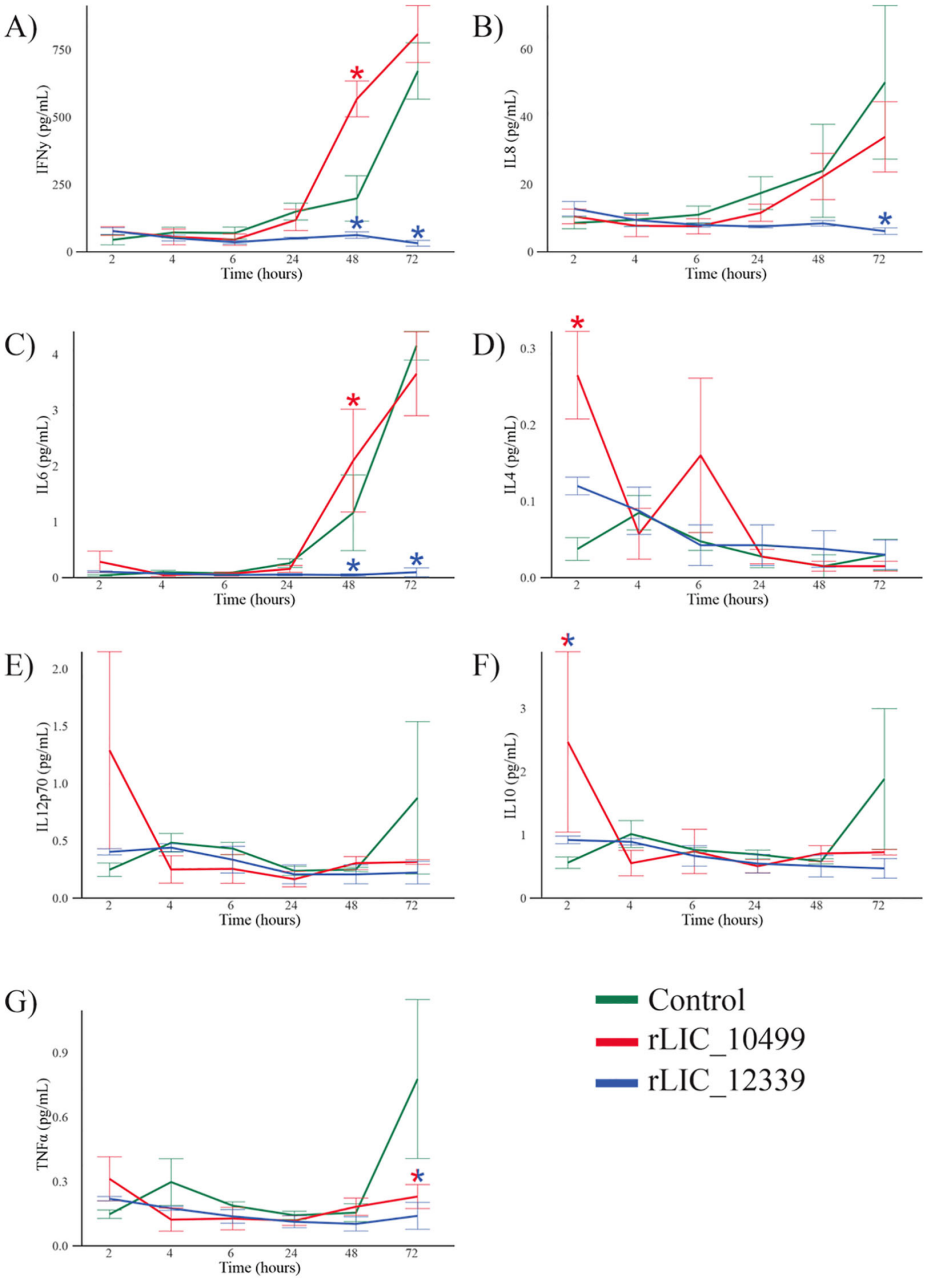
Multiplex analysis of cytokines produced by HEK-293T cells exposed to recombinant proteins rLIC_10499 and rLIC_12339. Twenty-four-well cell culture plates were seeded with 2.105 cells/well and incubated overnight at 37°C with 5% CO2 atmosphere. To wells, 1 µM of each recombinant protein with 1 µM aprotinin in DPBS were added and incubated at 37°C with 5% CO2 atmosphere for 72 h. As a control, DPBS supplemented with 1 µM aprotinin was used. At designated time points, aliquots of the reaction were taken and stored at – 80°C until further analysis. From A to G are the results of the cytokines evaluated IFN-g, IL-8, IL-6, IL-4, IL-12p70, IL-10 and TNF-α, respectively. (*) denotes statistically significance. Control (green); rLIC_10499 (red) and rLIC_12339 (blue).

Upon exposure to exogenous proteins, human cells initiate a robust response characterized by the production of cytokines and chemokines. This response represents a crucial mechanism in the interaction between host and pathogen. Variations in cytokine production can potentially dampen the immune response against invading bacteria, thereby facilitating their dissemination and survival within the host (67). To assess the cytokine profile elicited by recombinant proteins in epithelial cells, HEK293T epithelial cells were incubated with these proteins for periods ranging from 2 to 72 h. Analysis of inflammatory cytokine, such as, interferon-gamma (IFN-γ), interleukin-8 (IL-8), interleukin-6 (IL-6), interleukin-12 heterodimer (IL-12p70), tumor necrosis factor alpha (TNF-α) and anti-inflammatory cytokines, such as, interleukin-4 (IL-4) and interleukin-10 (IL-10) were subsequently conducted using a multiplex Luminex assay. As illustrated in **Figure 7**, a modest production of both inflammatory and anti-inflammatory cytokines was noted in epithelial cells stimulated with rLIC_10499. Notably, the production of IFN-γ by these cells started after 24 h of incubation, ultimately reaching significant levels by the 72-h mark (**Figure 7A**). Interestingly, the production of IL-8 and IL-6 occurred later compared to the inflammatory responses typically induced by pathogen-associated molecular patterns (68). Their production started after 24 h, with increasing concentrations up to 72 h (**Figures 7B, C**), which may suggest a strategy employed by the pathogen to establish infection before the activation of a robust innate immune response. The delay observed and the fact that the production peak of IL-8 and IL-6 was, respectively, one and two orders of magnitude lower than IFN-γ, taken together, may suggest an immune evasion strategy. A modest increase in the concentrations of IL-4, IL-12p70, IL-10, and TNF-α was detected at the initial stage of exposure (**Figures 7D-G**), with decreasing concentrations observed by 72 h. No cytokine production was observed following the stimulation of HEK293T cells with LIC_12399.

## Discussion

The ability of pathogenic *Leptospira* to adhere to host cells, invade host tissues, colonize, and resist innate immune attacks has contributed to its success in causing infection (13, 56, 69). In recent years, the study of hypothetical leptospiral proteins has led to the identification of several proteins involved in adhesion, colonization, invasion, and immune evasion (16, 54, 63, 70). Despite the identification of several leptospiral proteins, many proteins annotated in the *Leptospira* genome still have unknown functions. Here, we study two *L. interrogans* proteins, LIC_10499 and LIC_12339, which are conserved among pathogenic species, aiming to expand the understanding of *Leptospira* in host-pathogen interactions.

The protein LIC_10499 has a DUF1566 domain, which is named lcl c-terminal domain (71). Structurally, this domain is similar to C-type lectins that are involved in various biological processes, as cell-cell adhesion, immune response, and apoptosis (72). Moreover, modeled tertiary structure of this protein matches other pathogen proteins, such as sortase A from *Streptococcus pyogenes* (43), African swine fever virus core shell protein p15 (40), 3-dehydroquinate dehydratase, type II dehydroquinase and peroxidoxin BcpB from *Mycobacterium tuberculosis* (36–38), Norovirus 3CL protease (39), KlcA protein from *Bordertella pertussis* (42) and adenylyl-sulfate kinase from *Cryptococcys neoformans* (41). LIC_12339 possesses a DUF1561, belonging to the protein family named virulence modifying proteins (44, 73, 74). It has been reported that member of this family, when used as vaccine candidates in mice, were able to protect animals from severe leptospirosis and reduced bacterial load, indicating that this protein might be a promising vaccine candidate (75).

Different strategies are employed by pathogenic *Leptospira* to circumvent complement killing. The recruitment of regulators such as factor H (FH) and C4b-binding protein (C4BP) may help inactivating C3b convertase, thereby diminishing MAC formation and C3b opsonization. Another strategy is the binding of proteins to MAC components, such as C5, C7, and C9, which impairs the formation of an active MAC, pore formation and cell killing. Many leptospiral proteins have been characterized for their putative role in complement system inactivation, including Lsa23, LcpA, enolase and LigA and LigB proteins (10, 52, 76). It has been reported that TolC efflux protein binds FH, attenuates MAC deposition and enhances serum survival (70). The recombinant proteins rLIC_10499 and rLIC_12339 exhibited binding properties compatible to complement impairment, as they bind to FH, C4BP and C9, blocking the formation of C9 cylinders. Moreover, incubation of NHS with rLIC_10499 or rLIC_12339 led to increased survival of *E. coli* cells, suggesting that these proteins might be involved in complement immune evasion.

Another strategy used by *L. interrogans* during invasion is the acquisition of PLG and fibrinogen from human plasma, which aids its dissemination through the host. In its active form, PLA, it can degrade complement proteins, fibrin clots and adherent junctions’ proteins (57, 58, 77). The recombinant proteins tested here could sequester PLG from human plasma, making it available for conversion into PLA by PLG-activators. It has been demonstrated that PLA- associated *Leptospira* confers the bacteria proteolytic capabilities to the bacteria, allowing them to degrade ECM components, IgG and C3b (58). Binding to fibrinogen may also interfere with fibrin clot formation, facilitating the dissemination of the bacteria through tissues. The outer-membrane protein LIC_13086 inhibits fibrin clotting and sequesters C4BP, suggesting roles in both hemostatic disturbance and immune escape (63). Although several proteins have been described as fibrinogen binding proteins (62, 78, 79), only a few proteins are capable of inhibiting thrombin-catalyzed fibrin formation (60–63). This seems to be the case for both rLIC_10499 and rLIC_12339 proteins. While they showed the ability to binding fibrinogen, their inhibitory effect on fibrin formation was not observed.

During invasion, pathogenic bacteria must adhere to endothelial and epithelial cells, as well as proteins in the cell-cell interface, such as collagen, laminin and other ECM molecules, in order to disseminate. The mechanism involved in these interactions are not completely understood, as leptospires lack actin-modifying exotoxins and specialized secretion systems-molecules commonly used by other pathogens to disrupt the host cell cytoskeleton (80, 81). Thus, apparently pathogenic leptospires break this barrier by using alternative mechanism. Martinez-Lopez and collaborators (82) demonstrated alterations in cellular morphology and integrity of the cell layer when pathogenic leptospires were placed in contact with Ea.hy926 endothelial cells. In contrast, although pathogenic leptospires crossed MCDK epithelial cell layers more efficiently than the saprophyte *Leptospira*, no significant disruption was observed within the cell layers or the actin cytoskeleton (83), revealing that different strategies can be used by leptospires to interact with cells. Moreover, altering bacterial signal transduction pathways may upregulate virulence associated gene expression (84). Binding to cell receptors is another method used by bacteria to alter cell response to infection by altering cytokine production and barrier function (84). The adhesion of *L. interrogans* to cultured endothelial, fibroblast, kidney epithelial and macrophage cell lines has been investigated (64, 85, 86). It is known that adhesion is mediated, at least in part, by outer membrane proteins, and leptospires present a wide range of molecules involved in these interactions with ECM. However, to date, only LipL41, OmpL1, and rLIC_13555 have shown the ability to interact with endothelial and epithelial cells (13, 16). In addition to bind to ECM molecules, the protein rLIC_10499 was able to adhere to mammalian cells in both monolayers and suspension cultures. Furthermore, rLIC_10499 was capable of binding to surface receptors of HMEC-1, Ea.hy926 and HEK293 cells, while rLIC_12339 interact only to HEK293T cell receptors. Recent reviews on the cytokine response in human leptospirosis report a biphasic cytokine response pattern that correlates with the clinical disease phase. Severe leptospirosis develops a cytokine storm characterized by high levels of IL-6, TNF-α, and IL-10, resembling sepsis-like phenotypes (87). The cytokine profile produced by epithelial cells in response to *Leptospira* is unknown, but as epithelial cells respond to bacterial components by increasing the expression of pro-inflammatory mediators, a response was expected in the presence of leptospiral recombinant proteins. However, when HEK293T cells were exposed to rLIC_10499 a discrete production of cytokines was observed. The release of IL-8 after invasion of epithelial cells may serve as a signal for the initiation of an acute inflammatory response. Thus, this downregulation seems to affect local inflammatory response, which may assist the survival of invading bacteria. Additionally, Molinari et al. (88) observed a lack of inflammatory response in bovine endometrial epithelial cells and human monocytes exposed to heat-killed *Leptospira* or purified outer membrane preparations over 2, 12, and 24 h, suggesting that *Leptospira* may evade detection or trigger a different immune response.

In conclusion, our results showed that both rLIC_10499 and rLIC_12339 are proteins capable of interacting host complement molecules and mammalian cells. The investigation of *Leptospira* recombinant proteins interactions with these components reveals a sophisticated array of immune evasion strategies that certainly contribute to bacterial pathogenesis. The multifunctional nature of these proteins, their conservation across pathogenic species, and their roles in bacterial survival within the host make them attractive molecules for the study of their possible role as vaccine candidates.

## Data Availability

The original contributions presented in the study are included in the article/**Supplementary Material**. Further inquiries can be directed to the corresponding authors.

## Supplementary material

Supplementary Figure 1 Dose-dependent response of rLIC_10499 and rLIC_12339 to components of the complement system. Binding specificity was tested using dose-response curves in which components were fixed in microwell plates, followed by increasing concentrations of the recombinant proteins. The left and right panel refer to LIC_10499 and LIC_12339, respectively. **(A, B)** Factor H, **(C, D)** C4BP, **(E, F)** C7, **(G, H)** C8, **(I, J)** C9 while **(K)** C4b.

Supplementary Figure 2 Dose-dependent response of rLIC_10499 and rLIC_12339 to plasma components. Binding specificity was tested using dose-response curves; components were fixed in microwell plates, followed by increasing concentrations of the recombinant proteins. The left and right panel refer to LIC_10499 and LIC_12339, respectively. **(A, B)** fibrinogen, **(C, D)** plasma fibronectin, **(E, F)** PLG.
