# Supplementary figures and images for: The role of leptospiral proteins in immune evasion and inflammatory response stimulation in HEK293T cell monolayers
Source: Front Immunol. 2025 Dec 9;16:1689798. doi: 10.3389/fimmu.2025.1689798 (PMC12723034; doi:10.3389/fimmu.2025.1689798)

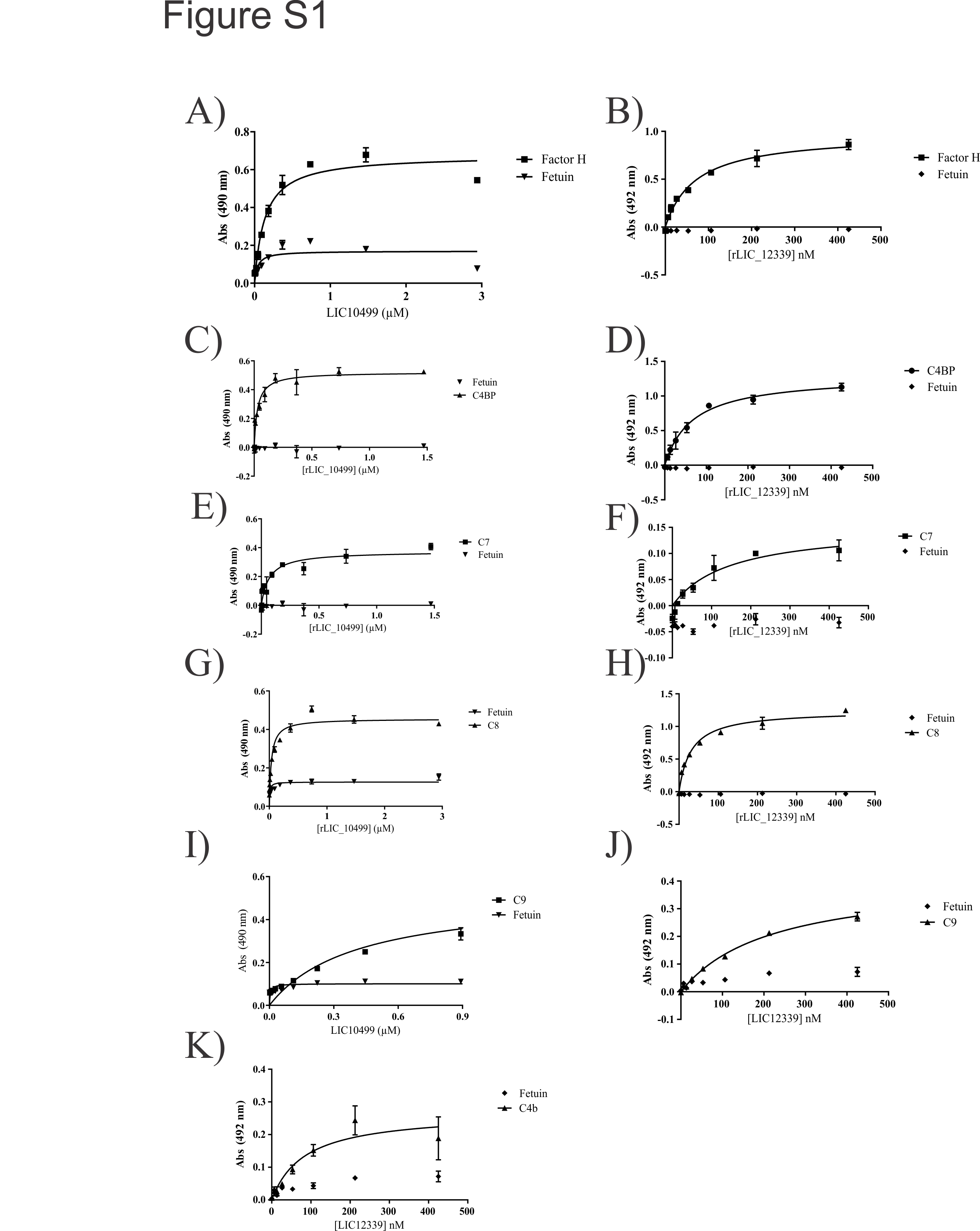

Supplement: Supplementary Figure 1 — Dose-dependent response of rLIC_10499 and rLIC_12339 to components of the complement system. Binding specificity was tested using dose-response curves in which components were fixed in microwell plates, followed by increasing concentrations of the recombinant proteins. The left and right panel refer to LIC_10499 and LIC_12339, respectively. (A, B) Factor H, (C, D) C4BP, (E, F) C7, (G, H) C8, (I, J) C9 while (K) C4b. [file Image1.tif]

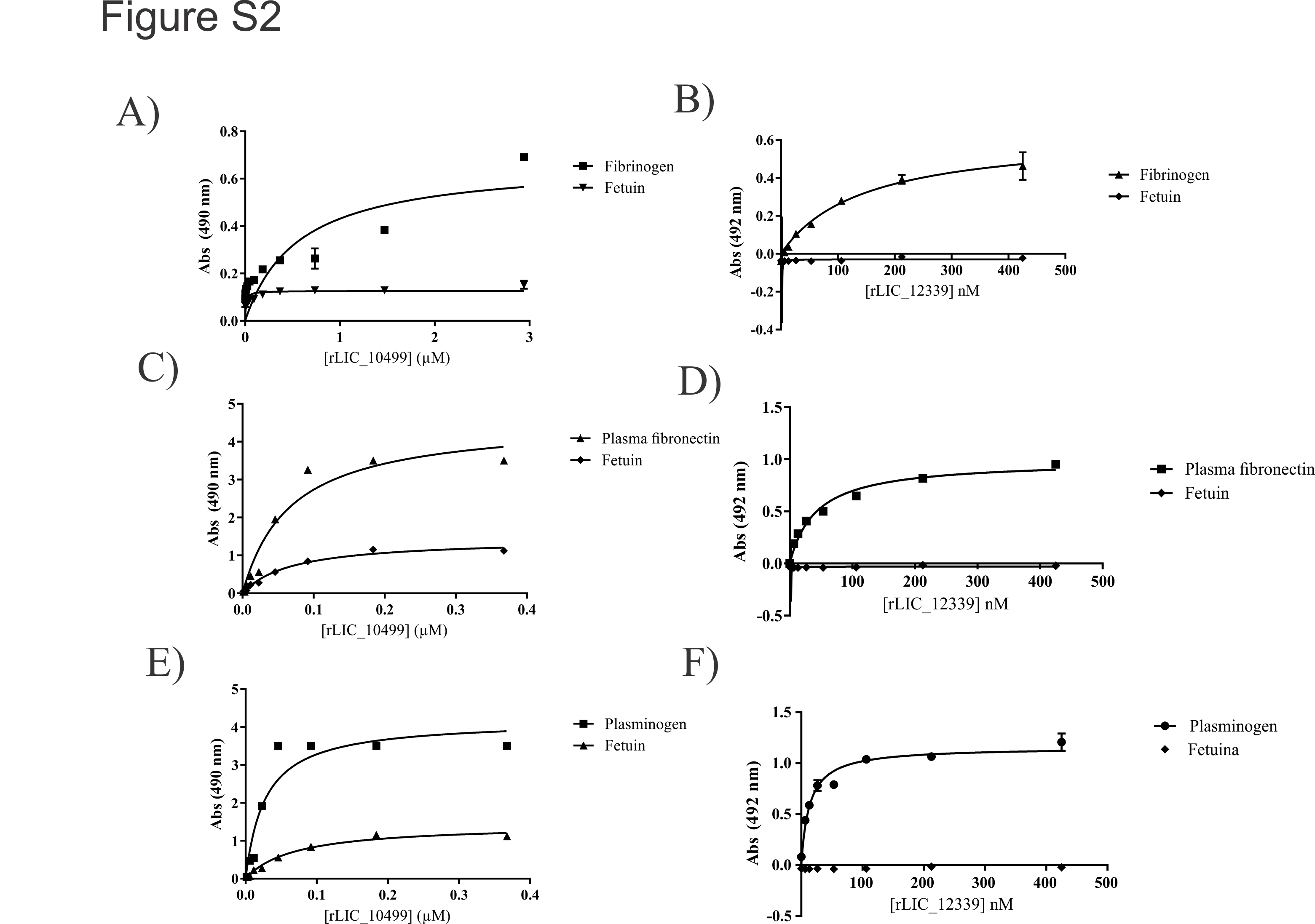

Supplement: Supplementary Figure 2 — Dose-dependent response of rLIC_10499 and rLIC_12339 to plasma components. Binding specificity was tested using dose-response curves; components were fixed in microwell plates, followed by increasing concentrations of the recombinant proteins. The left and right panel refer to LIC_10499 and LIC_12339, respectively. (A, B) fibrinogen, (C, D) plasma fibronectin, (E, F) PLG. [file Image2.tif]
